# Effect of incorporation of soy protein isolate and inulin on quality characteristics and shelf-life of low–fat duck meat sausages

**DOI:** 10.5713/ab.21.0530

**Published:** 2022-03-02

**Authors:** S. Moirangthem, S. K. Laskar, A. Das, S. Upadhyay, R. A. Hazarika, J. D. Mahanta, H. M. Sangtam

**Affiliations:** 1 Department of Livestock Products Technology, College of Veterinary Science, Assam Agricultural University, Khanapara, Guwahati-781022, India; 2 Department of Veterinary Public Health, College of Veterinary Science, Assam Agricultural University, Khanapara, Guwahati -781 022, India; 3 Department of Poultry Science, College of Veterinary Science, Assam Agricultural University, Khanapara, Guwahati-781022, India

**Keywords:** Duck Meat, Inulin, Sausage, Soy Protein Isolate

## Abstract

**Objective:**

Low fat duck meat sausages were prepared by replacing the fat in the formulations with soy protein isolate (SPI) and inulin to find the best formulation having superior shelf-life without affecting its quality attributes.

**Methods:**

Four sausage mix formulations were prepared viz.control (0% SPI and inulin), T_1_ (2.5% inulin), T_2_ (2.5% SPI), and T_3_ (2.5% SPI+2.5% inulin) replacing duck fat as per the recipe. Five batches of duck meat sausages of each formulation were prepared, and the final products were evaluated for physico-chemical, organoleptic, and microbiological qualities.

**Results:**

The % moisture and crude protein content of the sausages revealed an increasing trend (p<0.01) from control to the treated formulations, while the % total ash contents were found to be non-significant (p>0.05). On the contrary, the per cent ether extract decreased significantly (p<0.01) from the control to the treated groups. In terms of calorie value, control samples exhibited the highest values with a significant (p<0.01) regression from control to treated formulation, respectively. The colour profile study (*L*, a*, b*) of the formulations were found to be non-significant. Texture profile study in terms of springiness, cohesiveness, chewiness, and resilience revealed no significant difference in all the treatment groups except the hardness scores, which revealed a significantly (p<0.01) increasing trend from control to the treated formulations. The total viable count showed a significant decrease in the treated groups. However, there was a significant increase in the bacterial load during the storage till day 15th. The total viable psychrophilic bacterial count showed a significant (p<0.01) increase in bacterial load from day 5th to 15th day of storage. Colititre counts were negative for all the formulations until the 15th day of storage.

**Conclusion:**

The present study results may conclude that duck meat sausages could be prepared satisfactorily by replacing duck fat with SPI and inulin at the rate of 2.5% of each with superior quality attributes.

## INTRODUCTION

Meat is the most preferred food item in the diet of non-vegetarian consumers in India. Indian consumers generally prefer fresh meat cooked at home. However, urban consumers, especially the young generation, have changed their eating patterns, as they are more dependent on ready-to-eat types of meat products because of their busy lifestyles. The imposition of lockdown due to the COVID-19 pandemic has popularised ready-to-eat type online food delivery systems among modern meat consumers. At the same time, the consumer’s preference has increased for health-promoting foods and beverages to strengthen their immunity. Poultry meat is the most acceptable meat in India because of its lower price and the lower content of saturated fat than red meat species. Though chicken constitutes most poultry meat in India, duck meat is slowly gaining popularity due to its high nutritional value [1]. The aroma of duck meat is relatively more intense than other poultry meats. A study related to the sensory characteristics of duck meat found that the meat’s flavour is positively correlated with lipid content [2], and the higher fat content of duck meat may cause a more pungent flavour in duck meat.

Generally, various kinds of traditional food prepared from duck meat have grown over many countries. Peking roasted duck, Nanjing cooked duck, Zhangcha duck (China), Canard α l’Orange (France), Oritang (Korea), and gulaiitiak Ladomudo (Indonesia) are several traditional applications now found commonly. However, the traditional applications of duck meat cannot stimulate the rapid development of duck meat applications [3]. Contrary to other parts of the country, duck meat and eggs are much more preferred by the people of the North-eastern region of India. Farmers rear different indigenous breeds of ducks under traditional systems in this part of India. Therefore, duck meat has an advantage over commercial broiler chicken as it is considered near organic since there are no issues like antibiotics and growth-promoting hormones. Duck meat is a special delicacy, particularly in Assam. Many traditional dishes of duck meat in combination with ash gourd, black gram, banana flower, bamboo shoot etc., are prepared during festivals and ceremonies to entertain special guests. Production and processing of duck meat have tremendous scope for generating employment and boosting the farmers’ income in this region. Increasing consumers’ food literacy and health consciousness has forced the meat processing sector to develop healthy meat products. In the case of the most popular processed meat products, reducing fat is the first step to making them healthy [4]. However, it is a challenge to develop low-fat, reduced-fat meat products because reducing the fat content or improving the fatty acid profile results in the deterioration of technological quality attributes [5].

Soy protein is regularly used as a meat replacer due to its essential amino acids, whose composition (though slightly lower in quantity) is no different from meat [6]. Replacement of a more significant part of fat by thickening agents/meat extenders such as SPI provides lubricity, solubility and swelling. A fat mimic system influences texture properties in low-fat food [7].

The use of inulin in low-fat, fermented sausages produced a soft texture, tenderness, springiness, and adhesiveness nearly like conventional sausages [8]. Sausage is a processed meat product with elastic characteristics, and it is usually composed of beef and chicken meats. Sausage is generally prepared by mixing the minced meat with a binder and spices, and this formulation is then inserted into a particular casing. Sausage as a raw product is cooked by a steaming or smoking method [3]. Duck meat is the third most widely produced poultry meat globally after chicken and turkey [9]. Therefore, preparing duck meat sausages with reduced fat content incorporating soy protein isolate (SPI) and inulin has an excellent perspective for developing ready-to-eat healthy, low-fat meat products from local ducks in this region with good market potential.

## MATERIALS AND METHODS

### Preparation of products

The low-fat duck meat sausages were prepared as per a basic recipe (Table 1). The duck meat utilized in the present study was obtained from local ducks (Pati) of 8 to 10 months of age and 1.8 to 2.0 kg live weight collected from the nearby market (Beltola). The present research work was approved by the Institutional Ethics Committee of the Assam Agricultural University vide approval number: 770/GO/Re/S/03/CPCSEA/FVSc/AAU/IEAC/19-20/726 dated 23.12.2019. The birds were scientifically slaughtered with the electrical stunning method using an electrified water bath in the semi-mechanized poultry dressing unit of the Department of Livestock Products Technology, College of Veterinary Science, Assam Agricultural University, Khanapara. Then the carcass was deboned, and the visible fascia, cartilages, and all the separable fat were removed from the lean portion. The lean meat was obtained from the breast and thigh portion and then cut into small chunks that were vacuum packed separately in high-density polyethene bags, and stored at refrigeration temperature (−4°C±1°C). Subcutaneous, pelvic, and visceral fat were manually separated. The subcutaneous fat from the skin was separated by melting using a separating funnel and stored for later use.

Cellulose casing (21 mm diameter) was utilized to pre pare the sausages. Packaged SPI and inulin were purchased from a local supermarket. Whole dried spices were purchased from the local market and then washed, sun-dried and ground when necessary for using the same in sausage formulation. Condiments like garlic and gingers were procured from the local Beltola market and were cleaned/peeled and then chopped thoroughly. The condiment paste was prepared afresh for each batch of sausages. Spices and condiments were mixed as the given formula (Table 1) and used in the present study as per the recipe developed.

The deboned meat was minced twice in a mechanical meat mincer and thoroughly mixed with the curing ingredients, viz. salt (1.75%), sugar (0.5%), and sodium nitrite (150 ppm) before storing at refrigeration temperature for 24 hours. Afterwards, the sausage emulsion was prepared in a bowl chopper with the required amount of fat, non-meat ingredients, spices, and condiments. The emulsion thus prepared was divided into four parts. Excepting the control emulsion (C), the other three parts of emulsions were thoroughly mixed with 2.5% inulin (T_1_), 2.5% SPI (T_2_) and 2.5% SPI and 2.5% inulin (T_3_), respectively, before stuffing into the cellulose casings. Subsequently, the raw sausages were cooked at 80°C to 85°C for 45 minutes. Soon after cooking, the hot sausages were immersed in ice-cold water to prevent further cooking and to give a thermal shock to the surviving organisms. After cooling, the sausages were removed from the water and allowed to drain off the excess water. The chilled sausages were peeled and randomly packaged into food-grade high-density polyethene bags. The proximate quality parameters, colour and texture profiles were studied on the day of product preparation, and the microbiological quality traits were studied on the 1st, 5th, 10th, and 15th days of storage. Altogether, five batches of sausages were prepared for the present study.

### Proximate composition

The proximate composition of duck meat sausages was estimated as per the AOAC [10]. The crude protein content of the samples was determined by the Micro-Kjeldahl method by KEL PLUS KES 6L (Make: Pelican Equipment, Chennai, India), and fat contents were determined by Soxhlet methods (Make: Pelican Equipment, India; Model: KEL PLUS CLASSIC DX). The moisture content was assessed at 105°C under normal pressure by the drying method, whereas crude ash content was determined by placing the samples in a muffle furnace and operated at 525°C for 10 to 12 hours until white ash was obtained.

### Calorie value

Total calories in the cooked duck meat sausages were calculated based on 100 portions using the Atwater value for fat (9.00 kcal/g), protein (4.02 kcal/g), and carbohydrate (4.00 kcal/g).

### Colour profile

The colour profile of the product was evaluated with the help of a chromameter (Make: 3nh, Shenzhen ThreeNH Technology Co. Ltd. Shiyan, Shenzen, China; Model: NR 110) using (Commission Internationale de l’Eclairage (CIE) L*a*b* system where L* measure relative lightness or darkness, a* represents relative redness or greenesh and b* means relative yellowness or blueness.

### Texture profile

The texture profile of the sausage (hardness, springiness, chewiness, cohesiveness, and resilience) was determined with the help of a texture analyzer (Make: Stable Micro Systems, Godalming, Surrey, UK; Model: TA-HD plus).

Uniform samples (similar height) from each batch of products were used for the purpose. The test was conducted with a pre-test speed of 1 mm/s, test speed of 2.0 mm/s and post-test speed of 5 mm/s. The samples were pressed twice up to 50% of their original size with the help of an Aluminium Cylindrical probe (Make: Stable Micro Systems, UK; Model: SMS-P/36R).

### Microbiological qualities

#### Total viable count

Enumeration of the total viable plate count of the sausage samples was done in standard plate count agar medium by following the pour plate technique as described by APHA [11].

#### Yeast and mould count

The yeast and Mould Count of each sausage sample were enumerated following the method as described by APHA [12].

#### Total viable psychrophilic bacterial count

The total viable psychrophilic bacterial counts (TVPBC) of sausages were determined by the procedure described by the APHA [12].

#### Colititre

The colititre of the sausage samples were recorded following the ‘multiple tube technique’ as described by Harrigan and McCance [12].

### Statistical analysis

The data obtained in the study were analyzed statistically following the standard statistical method by employing SAS 9.3 software. Data were presented using basic descriptive statistics *viz*. mean and standard error. Comparison of different groups and storage days were analyzed using the two-way analysis of variance technique.

## RESULTS AND DISCUSSION

### Proximate composition

In the present study (Table 2), the moisture (%) of duck meat sausages incorporated with inulin (T_1_), SPI (T_2_) and SPI and inulin (T_3_) exhibited a significantly (p<0.01) increase trend from control to the treated formulations. Samples treated with inulin had higher moisture content which might be due to the presence of hydrophilic groups and the hygroscopic nature of inulin [13]. SPI treated formulations also revealed higher moisture content, probably due to their water-holding and water binding properties.

Similar to the findings of the present study, Akesowan [14] reported higher (p<0.05) moisture content with increased SPI levels in light pork sausages containing konjac flour. Alaei et al [13] reported that an enhanced level of inulin substitution in chicken sausages increased (p<0.05) the moisture content.

The treatment T _2_ (2.5% SPI) and T_3_ (2.5% SPI + 2.5% inulin) showed significantly higher (p<0.01) protein (%) content, probably due to the high content of protein in SPI while T_1_ (2.5% inulin) showed a slight increase compared to control, which was non-significant. Alaei et al [13] reported that an increased level of substituting fat with inulin enhanced the protein content in the chicken sausage samples. Akesowan [15] also revealed that the addition of SPI increased the protein content in light pork burgers. Similar findings were reported by Cengizand and Gokoglu [16], who observed that the addition of soy protein concentrate increased protein contents of frankfurters with 5% and 20% fat.

The per cent ether extract of duck meat sausages incorpo rated with SPI and inulin was significantly (p<0.01) lower than control and showed a decreasing trend in fat content from control to the treated formulations. The decrease in fat content of the sausages containing inulin is due to reducing fat and substitution with inulin. Similarly, the reduction in fat content in SPI treated samples is also because of fat replacement with SPI. Similar to the present study’s findings, Menegas et al [17] reported that the addition of inulin reduced the fat content compared to that in the control group. The fat content of chicken sausages was decreased with the increased levels of inulin substitution (p<0.05), as reported by Alaei et al [13]. The findings of the present study were also in agreement with Choi et al [18], who reported reduced fat in pork sausages formulated with chicory fibre and smoking treatment. Akesowan [15] also reported reduced fat content in light pork patties compared to the full-fat formulation of the product.

The total ash content of the treated formulations T _1_ (2.5% I), T_2_ (2.5% SPI), and T_3_ (2.5% SPI + 2.5% I) revealed no significant difference in comparison with the control product. Menegas et al [17] found no significant difference in ash content of dry fermented chicken sausages with inulin and corn oil. On the other hand, Cengiz and Gokoglu [16] reported that ash contents significantly (p<0.01) decreased with the addition of soy protein concentrate.

The calorie value recorded in control was 205.87±1.63 and in treated formulations T_1_, T_2_, and T_3_, the values recorded were 180.64±0.98, 180.99±1.48, and 165.94±2.42, respectively. The significantly low (p<0.01) calorie value in treated formulations might be due to the replacement of fat that contains high-calories and simultaneous substitution with SPI and inulin.

Nowak et al [19] reported that bologna-type sausages con taining 12% inulin had the lowest energy value among the treatment formulations. Calorie value was reduced both in conventional (23% fat) and reduced-fat sausages (10% fat) incorporated with inulin, as reported by Garcia et al [20]. Akesowan [15] reported that the calorie value of pork patties fortified with 2% SPI was reduced by 43.1% compared to the full-fat formulation of the product.

### Colour profile

The lightness (L) values of duck meat sausages incorporated with SPI and inulin revealed a non-significant reducing trend (Table 3) from control to treated products (T_3_). Redness (a) values also decreased non-significantly from control to T_3_. Decreased lightness (L) values in treated formulations might be due to fat reduction, which affects the colour of meat products by darkening. The decrease in the redness values might be due to the light cream colour of SPI and the whitish colour of inulin powder which replaced the dark colour of duck meat. Nowak et al [19] reported that the products tended to become darker and redder with increased inulin and decreased fat. The yellowness (b) value showed a non-significant increase from control to the treated formulations. Alaei et al [13] reported that substituting fat with inulin approximately increased the textural yellowness of chicken sausages. Das et al [21] also observed that the addition of soy granules and soy paste significantly (p<0.01) lowered the redness of goat meat patties and increased yellowness.

### Texture profile

The hardness value of treated products significantly (p<0.01) increased (Table 3) compared to the control. It might be due to the water-binding property and the protein available in SPI that contributed to protein gelation to modify the texture of the sausages [22]. Similarly, inulin-treated sausages had higher hardness values which might be related to the powder form of inulin added as the powder form tends to increase the hardness of meat products [20]. The findings of the present study corroborated sufficiently with the observations of Nowak et al [19] and Keenan et al [23].

The springiness values recorded for the treatment products with SPI and inulin showed no significant difference from the control. Choi et al [18] reported a slight decrease in the springiness value of restructured pork sausage with chicory fibre. However, Akesowan [15] noticed that springiness values of light pork burgers were higher than the control as the SPI level increased from 0.5% to 3%.

The treated sausages had higher cohesiveness values than the control; however, the difference was non-significant. Akesowan [15] reported that cohesiveness values of light pork burgers showed an increasing trend with the increase in SPI level from 0.5% to 3%. Similarly, Garcia et al [20] reported that reduced-fat sausages with inulin powder had slightly higher cohesiveness values than control.

The treated formulations had higher chewiness values com pared to the control. However, the differences were found to be non-significant. Keenan et al [23] observed increased chewiness values in comminuted meat products incorporated with inulin. Similarly, Akesowan [15] also reported higher chewiness values of light pork burgers than control as the SPI level increased from 0.5% to 3% (p<0.05).

The sausages treated with SPI and inulin revealed non-significantly higher resilience values than the control.

### Microbiological qualities

#### Total viable count

The duck meat sausages incorporated with SPI and inulin showed a significant (p<0.01) decrease in the total viable count (TVC) (Table 4) from control to treated formulations. Lower TVC in treated sausages might be due to the antimicrobial activity of SPI and inulin. Petkova et al [24] suggested that the antimicrobial activity of inulin acetates reveals a new aspect of their potential application against some plant and food-borne pathogens. Yeung et al [25] reported that the TVCs of the emulsified pork meatballs with the 2% whey protein and 6% isolated soy protein treatments were lower than that of the positive control group. Yim et al [26] stated that total plate bacteria and *Pseudomonas* counts of sausages were not significantly affected by fat level. Bacteria counts of sausages appeared to be unrelated to his study’s higher fat content. However, the increased moisture in products of this kind has not always been found to increase potential microorganism growth, perhaps because the effect of fat reduction on the microbiological status of meat products depends on numerous factors; these include processing variables (comminution, heat treatment, packaging, etc.), storage conditions (temperature, time, etc.) and formulation characteristics (meat source, added water, fat level, salt content, additives, etc.). An additional safety factor that should be considered is the use of some fat replacers [27].

However, there was a gradual increase in the count of TVC with the increase in the storage period. The increase in TVC of duck meat sausages might be due to multiplication of microorganisms during storage. Akesowan [15] reported increased total aerobic plate counts of control and 2% SPI light pork sausages during refrigerated storage. Biswas et al [28] observed that TVC values of duck patties increased significantly (p<0.01) with an increase in storage period at ambient and refrigeration temperature. Nowak et al [19] reported that the total aerobic plate count of bologna type sausages with inulin as fat replacer increased during the storage 23 days.

#### Total viable psychrophilic bacterial count

In the present study, the sausages incorporated with SPI and inulin showed a significant difference of TVPBC (Table 4) than control. Similar to the present study Sarteshnizi et al [29] observed that sausages with β-glucan and resistant starch showed lower total viable and psychrophilic count than control for 60 days and 30 days of storage, respectively.

However, there was a gradual increase in the count of via ble psychrophilic microorganisms with the increase in the storage period. It might be due to the growth and multiplication of psychrophilic organisms which came into contact during handling and storage. A significant increase in TVPBC values of duck patties with the increase in storage period was reported by Biswas et al [28]. Das et al [21] observed that psychrotrophic organisms were detected on day 10 and after that showed a significant (p<0.01) increase. A similar result was reported by Biswas et al [30] on duck meat sausage.

#### Yeast and mould count

In the present study, the yeast and mould counts (Table 4) were not detected until the 5th day of storage. However, they appeared on day 10 onwards and increased significantly (p<0.01) in all the treated products and the control. There was no significant difference between the treatment and control products. The detection of yeast and mould count on day 10 might be due to contamination during storage. There was a gradual increase in the yeast and mould count with an increase in the storage period.

The colititre value obtained for both control and treated samples with SPI and inulin, besides the sausages until the 15th day of storage, was nil. The absence of coliform count during the entire storage period might be due to heat treatment during cooking and better sanitary measures adopted during processing [21].

## CONCLUSION

Based on the results obtained on the various parameters studied in this investigation, it may be concluded that duck meat sausages can be prepared satisfactorily by partially replacing fat with SPI and inulin. Duck meat sausages of T_3_ formulation, with a combination of 2.5% SPI and 2.5% inulin, can be stored up to 10 days under refrigeration. Therefore, low-fat duck meat sausages, which are organoleptically acceptable, have higher protein content and are incorporated with dietary fibre, can be prepared successfully. However, further studies with more product formulations and a more significant number of samples with a longer duration of storage and with improved packaging system might be of immense value to draw a concrete conclusion and a recommendation for the best-suited formulation for a commercial venture.

## Figures and Tables

**Table 1:** Basic recipe of low-fat duck meat sausages with soy protein isolate and inulin

Name of ingredients	Control (%)	Treatment^1)^ (%)		

		T_1_	T_2_	T_3_
Lean meat	70	70	70	70
Duck fat (recovered from skin+visceral organs)	10	7.5	7.5	5.0
Soy protein isolate	0	-	2.5	2.5
Inulin	0	2.5	-	2.5
Spices	1.5	1.5	1.5	1.5
Condiments	3.45	3.45	3.45	3.45
Binder (Corn flour)	3	3	3	3
Ice cubes	10	10	10	10
Salt	1.75	1.75	1.75	1.75
Sugar	0.3	0.3	0.3	0.3
Sodium nitrite (ppm)	150	150	150	150
Total	100	100	100	100

ppm, parts per million.

1) T_1_, 2.5% inulin; T_2_, 2.5% SPI; T_3_, 2.5% inulin+2.5% SPI.

**Table 2:** Proximate composition of low-fat duck meat sausages incorporated with soy protein isolate and inulin (mean±standard error)

Parameter	Control	Treatment^1)^		

		T_1_	T_2_	T_3_
Moisture	60.09^a^±0.09	61.37^b^±0.12	62.24^c^±0.17	63.42^d^±0.23
Protein	21.07^a^±0.08	21.35^a^±0.21	23.39^b^±0.19	22.32^c^±0.14
Fat	11.60^a^±0.32	8.28^b^±0.16	8.39^b^±0.26	6.50^c^±0.21
Ash	3.05±0.04	3.03±0.02	3.00±0.05	2.90±0.06
Calorie value	205.87^a^±1.63	180.64^b^±0.98	180.99^b^±1.48	165.94^c^±2.42

SPI, soy protein isolate.

1) T_1_, 2.5% inulin; T_2_, 2.5% SPI; T_3_, 2.5% inulin+2.5% SPI.

N = 5.

a–d Mean with superscript bearing different alphabet row-wise differ significantly (p<0.01).

**Table 3:** Effect of incorporation of soy protein isolate and inulin on colour and texture profile of low-fat duck meat sausages (mean±standard error)

Parameter	Control	Treatment^1)^		

		T_1_	T_2_	T_3_
Colour profile				
L	59.18±1.48	59.57±1.96	57.85±2.42	58.60±1.39
a	9.12±1.48	8.93±1.42	8.62±1.45	8.67±1.41
b	15.92±0.91	16.35±0.84	16.82±0.82	16.56±0.54
Texture profile				
Hardness	886.15^a^±150.87	992.34^a^±245.86	1,300.91^a^±287.73	1,425.52^b^±224.37
Springiness	0.581±0.10	0.427±0.02	0.555±0.08	0.599±0.07
Cohesiveness	0.236±0.04	0.256±0.05	0.268±0.06	0.275±0.07
Chewiness	151.80±78.53	192.50±124.19	291.76±177.52	316.86±178.88
Resilience	0.071±0.01	0.171±0.08	0.087±0.02	0.097±0.03

SPI, soy protein isolate.

1) T_1_, 2.5% inulin; T_2_, 2.5% SPI; T_3_, 2.5% inulin+2.5% SPI.

N = 5.

a,b Mean with superscript bearing different alphabet row-wise differ significantly (p<0.01).

**Table 4:** Microbiological qualities low-fat duck meat sausages incorporated with soy protein isolate and inulin (mean±standard error)

Storage days	Control	Treatment^1)^		

		T_1_	T_2_	T_3_
Total viable count (log cfu/g)				
Day 1	2.32^a^^A^±0.01	2.24^a^^A^±0.00	2.25^a^^A^±0.01	2.14^b^^A^±0.01
Day 5	3.46^a^^B^±0.03	3.34^b^^B^±0.03	3.34^b^^B^±0.03	3.23^^c^^^B^±0.02
Day 10	4.65^a^^C^±0.01	4.55^b^^C^±0.01	4.54^bc^^C^±0.01	4.46^^c^^^C^±0.01
Day 15	5.35^a^^D^±0.01	5.24^b^^D^±0.01	5.24^b^^D^±0.01	5.16^b^^D^±0.02
Yeast and mould count (log cfu/g)				
Day 1	-	-	-	-
Day 5	-	-	-	-
Day 10	1.86^a^^A^±0.00	1.84^a^^A^±0.00	1.85^a^^A^±0.00	1.79^a^^A^±0.02
Day 15	2.16^a^^B^±0.00	2.14^a^^B^± 0.01	2.15^a^^B^±0.00	2.13^a^^B^±0.01
Total viable psychrophilic bacterial count (log cfu/g)				
Day 1	-	-	-	-
Day 5	2.59^a^^A^±0.01	2.47^b^^A^±0.02	2.48^b^^A^±0.01	2.37^c^^A^±0.02
Day 10	3.28^a^^B^±0.01	3.20^b^^B^±0.01	3.20^b^^B^±0.02	3.14^b^^B^±0.00
Day 15	3.84^a^^C^±0.01	3.78^b^^C^±0.02	3.79^b^^C^±0.01	3.72^b^^C^±0.02

SPI, soy protein isolate.

1) T_1_, 2.5% inulin; T_2_, 2.5% SPI; T_3_, 2.5% inulin+2.5% SPI.

N = 5.

a–c Mean with superscript bearing different alphabet (small) row-wise differ significantly (p<0.05).

A–D Mean with superscript bearing different alphabet (capital) column-wise differ significantly (p<0.01).
